# The Effects of Minie Balls on Bone

**Published:** 1864-06

**Authors:** E. Lloyd Howard

**Affiliations:** Surgeon 27th Reg't N. C. T., Cooke's Brigade, Army Northern Virginia.


Art. VI.—*The Effects of Minie Balls on Bone.* By E. Lloyd Howard, Surgeon 27th Reg't N. C. T., Cooke's Brigade, Army Northern Virginia.

Since the introduction of the Minie ball into warfare, surgical writers, with entire unanimity, have agreed that wounds of bony structures, inflicted by this missile, are characterized by extensive fissuring and comminution, such as was rarely, if ever seen, when the old smooth-bore musket was the weapon of the soldier. In every recent work on military surgery, we are told that the adoption of the improved weapon has "revolutionized" this branch of the science; and these writers would have us believe that this supposed extensive fissuring of bone is a new element, which should materially modify our prognosis and treatment of this class of injuries.

This doctrine I believe to be false in theory, and directly contrary to the teachings of experience.

The difference in the effects of the two balls depends upon their different rates of velocity; consequently, we should naturally expect that that missile having the greater degree of force, would cause the lesser splintering of bone—just as we see a bullet from a rifle pierce a pane of glass, leaving a clean, round orifice, without radiating fractures, while the same projectile thrown with *less* velocity, as from the hand, will shatter the glass into fragments.

In a late publication, "a Manual of Military Surgery," prepared for the use of the Confederate States Army, by order of the Surgeon-General, we find, in the chapter on gunshot wounds, the following passages:

"When a cannon ball, *at full speed*, strikes, in direct line, a part of the body, it carries away all before it. * * * If it be part of one of the extremities which is thus removed, the end remaining attached to the body presents a stump with nearly a level surface of darkly contused, almost pulpified tissues. * * * Minute particles of bone will be found among the soft tissues on one side, *but the portion of the shaft of the bone remaining in situ is probably entire.*" "*In ricochet-firing, or in any case where the force of the cannon-shot is partly expended*, the extremity, or portion of the trunk, may be equally carried away; but the laceration of the remaining parts of the body will be *greater*. The surface of the wound will be less; even muscles will be separated from each other and hang loosely, offering, at their divided ends, little appearance of vitality; spiculæ of larger size will probably be found among them, *and the shaft may be found shattered and split far above the line of its transverse division.*"

This description of the difference in wounds, caused by balls at great and lesser velocity, is well drawn and true to nature. But, though admitting this difference in the case of *cannon-balls*, the writer alleges a directly opposite condition to exist in wounds from *musket balls*. At page 41 occurs the following: "*A rifle bullet which splits up a long bone into many longitudinal fragments, inflicts a very much more serious injury than the ordinary fracture effected by the ball from the smooth-bore musket.*"

Why should not here, also, the projectile of more rapid flight produce the cleaner section, just as has been admitted to be the case with cannon-shot? It may be said that there are other points of difference between the two missiles than that of velocity—as the conical form, and the rotary motion of the Minie bill—two features wanting to that of the smooth-bore.

How the rotary motion could have any influence in producing the fissuring, is not apparent The *form* of the conical ball gives it, it is asserted, the peculiar power of the wedge, and that it is by virtue of this power that it produces the supposed fissuring. That this wedge-like shape gives the ball easier passage through the air or other obstacles of slight molecular tenacity, which it may encounter in its flight, may be true. On meeting with an object of greater density and of fibrous structure, that the fibres should rather be separated in their length than torn across by such a missile, giving it passage through the rent, would seem a natural supposition, and such separation be apt to extend beyond the immediate point struck. But that such is not the fact in the case under consideration, experience abundantly proves.—When a conical ball strikes a plank, we find as little splitting of the wood as from a round ball; and here the fibrous character of the substance is far more marked than in the case of bone.

That this wedge power is not exercised, is partly due to the fact that immediately, on contact, the leaden missile is somewhat flattened out of its original shape, (even by substances of less density than its own,) but principally because *great velocity robs the wedge of its peculiar force, which is essentially slow in its action.*

Wounds from the smooth-bore musket are now but rarely seen by the army surgeon, but we often have presented to us injuries of a similar character from fragments of shell, balls from spherical case shot, and the rifle ball, whose force has been partly expended by distance, etc., and the similarity of this last to wounds from round balls is an additional argument, if any were needed, against the theory of wedge-action. In these cases I have almost invariably found more extensive comminution than in those where the limb has been pierced by the Minie ball in full flight.

Where you have a ball impacted in the shaft of a bone, you will generally find fissures extending for a considerable distance both upwards and downwards. I have frequently seen this in wounds from the round leaden ball, musket-size, from spherical case, whose velocity is seldom sufficient to carry it entirely through a limb.

On the other hand, where we have a limb pierced by the Minie ball, with the orifices of entrance and exit of the same size and appearance—which is an indication that the ball passed at great velocity, with unimpaired force, we may feel assured that a clean cut has been made through the bone, and that there is no great fissuring around the immediate vicinity of the point struck. In the large number of amputations and resections for wounds of this nature, which I have witnessed upon the different battle-fields, rarely, if ever, have I seen fissures extending along the shafts of the bone.

Both the Minie and round ball, in passing through a bone, destroy every thing in their paths; but the missile of the *lesser* velocity exerts its destructive influence over a *wider sphere around that path* than that of the *greater speed*, whose injurious effects *are confined to its immediate track*, just as I have before illustrated, in the case of the pane of glass.

